# Deadwood Mitigates Declines in Soil Food‐Web Energy Fluxes in Canopy Gaps

**DOI:** 10.1111/gcb.71197

**Published:** 2026-11-08

**Authors:** Yan Zhang, Zheng Zhou, Gennuo Wang, Huimin Yi, Sarah L. Bluhm, Ajuan Zhang, Johanna E. Noske, Bin Xiao, Stefan Scheu, Melanie M. Pollierer, André Junggebauer

**Affiliations:** ^1^ J.F. Blumenbach Institute of Zoology and Anthropology University of Göttingen Göttingen Germany; ^2^ Ecology of Tropical Agricultural Systems University of Hohenheim Stuttgart Germany; ^3^ German Centre for Integrative Biodiversity Research (iDiv) Halle‐Jena‐Leipzig, Puschstraße Leipzig Germany; ^4^ Institute of Biology Leipzig University, Puschstraße Leipzig Germany; ^5^ Department of Soil Zoology Senckenberg Museum of Natural History Görlitz Görlitz Germany; ^6^ Centre of Biodiversity and Sustainable Land Use University of Göttingen Göttingen Germany; ^7^ Julius Kühn‐Institute Quedlinburg Germany

**Keywords:** carbon cycling rate, energy flux, food web architecture, forest disturbance, forest management, soil animals, trophic interaction

## Abstract

Across forests worldwide, frequent climate extremes and intensified timber harvest are reshaping forest structure by increasing tree mortality and canopy opening, threatening terrestrial biodiversity and ecosystem sustainability. In unmanaged forests, natural disturbances create canopy gaps and leave substantial deadwood on site, whereas timber harvesting creates gaps through the removal of living trees, thereby limiting the accumulation of deadwood. However, how these disturbances and management practices affect ecosystem functioning, especially of the belowground system, remains little understood. Here, we reconstructed 116 soil animal food webs in a full‐factorial experiment manipulating forest gaps and deadwood addition. We quantified energy fluxes of soil food webs as an integrative measure of belowground ecosystem functioning and investigated their network architecture 2 years after establishment of the experiment. Forest gaps reduced total food web energy fluxes by 13.4%, decreased network connectance, and shifted energy flow towards the bacteria‐dominated “fast” channel, indicating simplified soil food webs, impaired soil functioning, and reduced energy retention belowground. However, compared to gaps without deadwood, the presence of deadwood in gaps increased total energy flux by 37.4%, primarily through enhanced fungivory and wood‐feeding pathways, and restored network connectance and cycling rates to levels comparable to those in closed‐canopy forests. Our results demonstrate that deadwood acts as a key structural component that mitigates the detrimental effects of canopy gap formation on belowground ecosystem functioning, underscoring deadwood retention after timber harvest as a practical strategy to safeguard soil functioning in a more disturbance‐prone future.

## Graphical abstract

Deadwood mitigates declines in soil food‐web energy flux and network structure in canopy gaps. Blue and red denote negative and positive effects, respectively; gap effects are shown relative to undisturbed control plots, and deadwood + gap effects relative to gap plots. Energy flux refers to total soil food‐web energy flux integrated across 13 major soil animal groups. Basal resources of soil food webs are not shown. The undisturbed control and treatment of deadwood added are omitted for clarity.

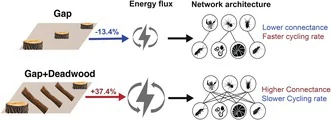

## 1 Introduction

Forests are central to sustainable development because they mitigate climate change through carbon storage and sustain biodiversity and ecosystem services (Brockerhoff et al. 2017; Guo et al. 2019; IPCC 2021). However, forest sustainability is increasingly challenged worldwide by intensified timber harvesting and more frequent climate extremes and pest outbreaks, all of which elevate tree mortality and result in widespread canopy opening (Pugh et al. 2019; Seidl et al. 2017; Turner 2010). Such canopy loss has accelerated globally, accounting for 12% of annual carbon turnover (Pugh et al. 2019), and has become particularly pronounced in central Europe due to a legacy of intensive management combined with recent drought events (Grünig et al. 2026; Neumann et al. 2017; Senf et al. 2018). Given that forest soils hold the majority of ecosystem carbon, management‐ and disturbance‐driven canopy loss may have disproportionate effects on long‐term carbon storage by reshaping belowground processes (Scharlemann et al. 2014). In addition to canopy gap formation, the accumulation of deadwood represents another pervasive structural consequence of tree mortality (Franklin et al. 2002; Seidl et al. 2017), and which of these consequences dominate depends on management scenarios. In unmanaged forests, dead trees are left to decay, corresponding to the situation of natural disturbances, whereas they are typically removed during wood harvesting in managed forests, causing large‐scale canopy gaps but limited deadwood retention. This divergence raises a widespread but poorly resolved gap in our understanding of how canopy gaps and deadwood, as key structural features associated with tree mortality, jointly influence ecosystem functioning, especially belowground.

Most of the primary production of forests is not consumed by herbivores but enters the belowground system, mainly as detritus (Crawley 1997; Polis and Strong 1996) and root exudates (Högberg et al. 2001; Pollierer et al. 2007), which may contribute as much as 50% of the daily assimilated carbon or account for up to 30% of annual net primary production (Collalti et al. 2020; Guenther 2002). These inputs sustain a large share of global diversity and, via soil food webs, drive litter decomposition, and nutrient cycling, thereby supporting the aboveground vegetation and whole‐ecosystem functioning (Anthony et al. 2023; Moore et al. 2004; Ruess et al. 2025). Forest gaps and deadwood strongly modify the availability of detrital resources as well as the physical environment, and thereby result in the re‐assembly of soil animal communities. For example, forest gaps tend to reduce densities of drought‐ and heat‐sensitive soil fauna, including, for example, earthworms, collembolans, and oribatid mites (Junggebauer et al. 2024; Reed et al. 2023; Zhang, Zhang, et al. 2025), whereas deadwood often promotes the diversity of soil organisms, especially saprotrophic ones, by improving habitat conditions and providing additional detrital food resources (Lassauce et al. 2011; Perreault et al. 2023; Seibold et al. 2015). In addition, decomposition generally accelerates at higher temperature and deadwood may decompose particularly rapidly in canopy gaps (Griffiths et al. 2021; Perry et al. 2018). This suggests that deadwood inputs have disproportionately strong effects on detrital resources and soil biota in canopy gaps, while existing evidence remains largely taxon‐specific. Consequently, it is still unclear how canopy gaps and deadwood rewire soil food webs and alter belowground ecosystem functioning.

In soil food webs, energy is transferred from basal resources through detritivores, microbial feeders and herbivores to higher‐level predators, thereby linking trophic functions, that is, herbivory, microbivory, and predation (Baiser et al. 2011; Buzhdygan 2023; Krishna and Mohan 2017). Embedding these transfers in an explicit network framework reveals how energy is routed among basal resources and consumer trophic groups (Coux et al. 2016; Kéfi et al. 2015; Schwarz et al. 2017), and provides a common basis for evaluating ecosystem multifunctionality (Henneron et al. 2026; Potapov 2022). For example, soil animals relying relatively more on bacterial resources are generally associated with faster‐cycling detrital energy channels, whereas greater reliance on fungal resources tends to be linked to slower‐cycling pathways based on more recalcitrant organic matter (Lu et al. 2025; Rooney and McCann 2012; Wenglein et al. 2026). Accordingly, warmer conditions following gap formation may shift consumer energy use towards “faster” bacterial‐dominated pathways (Coleman et al. 1983; Yu et al. 2022), whereas deadwood may strengthen “slower” fungal‐dominated pathways and the associated trophic links to soil animals (Coleman et al. 1983; Stokland et al. 2012). Therefore, energy‐flux approaches allow disentangling ecosystem energy budgets and food‐web architecture but have rarely been used to investigate the consequences of forest management practices.

Here, we used a large‐scale, full‐factorial field experiment across 29 temperate forest sites in three regions of Germany (northern, central, and southern) to investigate how canopy gap formation and deadwood addition reshape soil food‐web energy fluxes and network architecture. At each site, we established four treatments including forest gap formation (gap vs. closed canopy) and deadwood addition (with vs. without) and reconstructed 116 soil animal food webs based on 13 major consumer groups, such as earthworms, spiders, centipedes, and beetles. The three regions differ in climate and geological history, allowing us to generalize the results across environmental gradients and large spatial scales. We tested three hypotheses:

*Forest gaps reduce total food web energy flux due to reduced plant‐derived resources*, i.e., *leaf litter input and labile root exudates below the ground* (Högberg et al. 2001; Zhang et al. 2024).

*Deadwood increases food web energy flux by providing additional detritus and buffering microclimate fluctuations, with particularly strong effects in canopy gaps* (Graf et al. 2022; Perreault et al. 2023).

*Forest gaps favor bacterial‐dominated energy channels by disproportionately reducing fungal resources through reduced root‐derived C* (Arunachalam and Arunachalam 2000), *while deadwood addition favors the fungal‐dominated pathway through the addition of woody material and associated buffering of adverse abiotic conditions* (Bani et al. 2018; Lassauce et al. 2011; Perreault et al. 2021).

## 2 Material and Methods

### 2.1 Study Sites and Experimental Design

The study was conducted within the framework of the Biodiversity Exploratories (https://www.biodiversity‐exploratories.de/en/), a long‐term and large‐scale platform for biodiversity research across three geographical regions of Germany (Fischer et al. 2010). In total, there were 29 sites across three regions. Twelve sites were located in northern Germany (Schorfheide‐Chorin), comprising three beech, three pine, three pine‐beech mixed, and three oak forests; nine sites were located in central Germany (Hainich‐Dün), comprising five even‐aged and four uneven‐aged beech forests; and eight sites were located in the southern Germany (Schwäbische Alb), comprising three beech forests, three spruce forests, and two mixed spruce‐beech forests. The northern region is a young glacial landscape with elevations of 3–140 m and annual precipitation of 500–600 mm. Soils are cambisols with a pH of ~3.3. The central region is located at elevations of 285–550 m and receives annual precipitation of 500–800 mm. Parent rock is limestone and the soils are rich in clay with a pH of ~4.3. The southern region is situated at an elevation of 460–480 m, with an annual average precipitation of 700–1000 mm. Parent rock is limestone with loamy and clay‐rich soils of a pH of ~4.9.

In early spring 2020, a forest gap and deadwood addition experiment was established at 29 sites across three study regions. At each site, four treatments (plots) were established: closed canopy without deadwood (*Control*), canopy gap without deadwood (*Gap*), closed canopy with deadwood (*Deadwood*), and canopy gap with deadwood (*Gap + Deadwood*), yielding a total of 116 plots. The control plot spanned 100 × 100 m, while the other plots spanned 71 × 71 m. Canopy gaps were established by felling trees in a circular area of on average 31.0 ± 3.2‐m diameter, defined by the mean top stand height at each site (25–37 m). This corresponded to an estimated 33.2 ± 11.4 m^3^ of stem volume removed per gap. Aboveground biomass of cut trees was removed from the plots while belowground root material remained in the soil. Plots with deadwood addition received fresh deadwood from the felled trees of the adjacent gap treatments. The trees were cut into 4–5‐m logs and distributed to the two deadwood treatments in predefined amounts. The target volume of deadwood was calculated from the standing tree volume of each plot using the formula 0.25 × standing tree volume + 50, resulting in the added deadwood volume ranging from 121.3 to 212.7 m^3^ ha^−1^ (average 156.9 ± 21.4 m^3^ ha^−1^) (Staab et al. 2022). Tree logs were moved into deadwood treatments using machinery (harvesters and forwarders), horses, or manually, with minimum disturbance. Within each site, plots were located in similar topographic and exposure conditions and were at least 100‐m apart.

After gap formation, the understory vegetation developed vigorously. Plant communities were dominated by grasses (mainly *Brachypodium sylvaticum* ), sedges (mainly *Carex sylvatica* ), shrubs (mainly *Rubus* species), and herbaceous species (mainly *Urtica dioica* and *Oxalis acetosella* ), as well as tree seedlings (mainly of *Fagus sylvatica* , *Fraxinus excelsior* , and *Picea abies* ). For details, see previous work (Zhang, Zheng, et al. 2025).

### 2.2 Sampling, Extraction, and Determination of Soil Fauna

Sampling was conducted in May 2021, about 2 years after establishment of the experiment. At each of the 29 sites, soil cores were taken in a 3 × 3‐m subplot of each of the four treatments. The 3 × 3‐m subplot was located in the center of each experimental plot, and soil cores were collected randomly within this subplot. In the deadwood treatments, sampling points were typically located approximately 40 cm from logs in the deadwood treatments. Two soil cores of diameters of 20 and 5 cm were taken from a total of 116 plots (29 sites × 4 treatments). The 20‐cm soil cores were used to extract macrofauna, that is, Araneae (spiders), Chilopoda (centipedes), Diplopoda (millipedes), Coleoptera (beetles), Diptera larvae, Isopoda (woodlice), Lumbricidae (earthworms) and Pseudoscorpionida (pseudoscorpions). The 5‐cm soil cores were used to extract mesofauna, including Collembola, Oribatida, Mesostigmata, Diplura, and Symphyla. The animals were extracted separately from the litter layer and the upper 5 cm of soil using high‐gradient heat extractors (Kempson et al. 1963; Macfadyen 1961). Soil animals were preserved in 70% ethanol until taxonomic identification. In central and southern sites, Lumbricidae were additionally collected after applying 10 L of a 10% mustard solution to holes where the 20‐cm cores had been taken (Eisenhauer et al. 2008). At the northern sites, Lumbricidae were collected exclusively by heat extraction from the 20‐cm cores, as the earthworm communities in this region are heavily dominated by epigeic earthworm species (Pollierer et al. 2025), likely due to acidic soil conditions. Therefore, soil‐core sampling and heat extraction were considered sufficient.

In total, 13 soil animal groups were included for reconstructing soil food webs. Macrofauna, including Lumbricidae, Chilopoda, Diplopoda, and Isopoda, were identified to species level, using keys of Sims and Gerard (1985) for Lumbricidae, Andersson et al. (2006) and Klausnitzer (2019) for Chilopoda and Diplopoda, and Schaefer (2018) for Isopoda. Coleoptera were classified into four trophic groups, that are, Elateridae (detritivores), Staphylinidae (predators), Curculionidae (herbivores), and others. Araneae, Diplura, Diptera, Pseudoscorpionida, and Symphyla were not identified further. Mesofauna, that is, Collembola, Oribatida, and Mesostigmata, were counted, measured and determined to species level using the keys of Weigmann (Weigmann 2006) for Oribatida, Hopkin (1997) for Collembola and Karg (1989) for Mesostigmata. The body size for all individuals (both adults and larvae) was measured for each group and used to calculate biomass. Additionally, three 5‐cm diameter soil cores were taken from each experimental plot, and each the three litter and three underlying 0–5 cm mineral soil samples were pooled and stored at −20°C for analyses of microbial biomass C and PLFA‐based microbial community composition. These variables were subsequently used as environmental predictors of soil food‐web energy flux.

### 2.3 Biomass Estimation

We estimated biomass from fresh body mass using taxon‐specific allometric equations (Table S1). For Collembola, Oribatida and Mesostigmata, body length and width were measured for all individuals (adults and juveniles) when there were < 10 individuals per species in a sample; when ≥ 10 individuals were present, 10 were chosen at random for measurement. The mean body size of each species in each sample was then used to calculate body mass. For all other taxa, with the exception of Symphyla, length and width of every individual were measured and converted to body mass. Then, the biomass of each taxon (g m^−2^) was calculated by multiplying the site‐specific body mass by its density. For Symphyla, biomass was calculated using region‐specific mean fresh body masses (northern, central, and southern) derived from previous measurements in the same regions because individual body‐size measurements were unavailable. Excluding Symphyla did not qualitatively alter the energy‐flux results, but we retained this group to provide a more complete representation of the soil food web.

### 2.4 Estimation of Metabolic Rates

Following Ehnes et al. (2011), mean metabolic rates for each taxon in each sample were calculated using the Boltzmann–Arrhenius allometric formula *I* = *i*_*0*_ × FM^*a*^ × exp.(−*E*/*KT*), where *I* is metabolic rate (J/h), *i*_*0*_ a normalization factor, *a* the allometric exponent, FM the individual fresh body mass (mg), *E* the activation energy, *K* the Boltzmann constant and *T* the site‐ and treatment‐specific temperature in Kelvin. Temperature was based on hourly measurements taken 20 cm above soil surface and averaged over 3 months in spring (March, April, and May 2021), that is, the period soil animals were exposed to before sampling. Total taxon‐specific metabolic rates (J h^−1^ m^−2^) in each sample were calculated by multiplying mean metabolic rates and taxon‐specific density (ind. m^−2^).

### 2.5 Soil Food Web Reconstruction

We reconstructed soil food webs using a multichannel approach following Potapov (2022). All animal taxa were first aggregated into 16 broad taxonomic/functional groups and then assigned to trophic roles based on (1) feeding preferences, (2) predator and prey functional traits and (3) life stage. For each of the 16 taxonomic/functional groups we specified feeding preferences for plants (P), algae (A), living roots (L), wood detritus (W), bacteria (B), fungi (F), soil organic matter (S) and animals (predation). Overall, we defined eight resource categories, which also served as prey categories in the interaction matrix and formal statistics. Preferences were coded as 1 (main resource), 0.5 (supplementary resource) or 0 (not used). Predation strength between predators and prey was further adjusted using predator traits (e.g., venom, hunting mode, habitat filtering) and prey defense (e.g., physical protection, defensive metabolites, agility). More details on the taxonomic grouping, feeding preferences, functional traits and vertical distribution are provided in Table S2.

We inferred weighted trophic interactions among nodes in each food web using the following steps:

1. All potential consumer‐resource links were first assigned among the nodes following Potapov (2022). Feeding preferences were coded as 1 (main food), 0.5 (supplementary food), and 0 (no link).
2. For each consumer node, we then rescaled the preferences to obtain relative diet proportions, assuming that supplementary resources are consumed five times less than main resources. For omnivores feeding on both animals and basal resources, we set 50% of intake to predation and 50% to all other basal resources. Diet proportions for each consumer were summed to 1 and served as initial interaction weights.
3. Predation strengths were further modified using an optimum predator–prey mass ratio, assuming predators are typically 100 times heavier than their prey (Brose et al. 2008). For each predator group, we used the mean and standard deviation of body mass from all collected specimens (27,938 individuals) to derive the expected body‐mass distribution of optimal prey. Predator traits (e.g., parasitism, cooperative or pack hunting, venom, specialized mouthparts) were used to broaden this distribution towards similar‐sized or larger prey. Conversely, earthworms may consume much smaller prey by ingesting soil particles colonized by microorganisms. We then quantified the overlap between the optimal prey‐size distribution of each predator group and the observed body‐mass distributions of all potential prey groups; this overlap was used as a proxy for body mass‐based interaction strength.
4. We reduced predation strength according to prey defensive traits. For each prey group we assigned a protection coefficient based on its suite of defenses (e.g., physical protection, defensive metabolites, agility): 1 (no protection), 0.7 (moderate protection) or 0.4 (strong protection). These coefficients were used to down‐weight the corresponding predator–prey links.
5. Finally, we accounted for the vertical distribution of taxa. We distinguished four vertical layers: epedaphic (litter surface), hemiedaphic (litter layer), and euedaphic (mineral soil). For each trophic group, we assigned the proportion of its biomass in each layer. The layer with the highest biomass was set to 1, and proportions in the other layers were scaled relative to this maximum; very small non‐zero values (< 0.05) were rounded to 0.05 to retain occasional occurrences. Based on these vertical distribution values, we calculated pairwise Bray–Curtis dissimilarities among all consumer nodes. The complement dissimilarity (1‐dissimilarity) was used as a measure of spatial overlap and served as an additional weight on interaction strength.

These components, that are, feeding preferences, body mass matching, predation traits, prey protection and vertical overlap, were combined using an adjacency matrix to obtain weighted predator–prey interactions for each soil food web.

### 2.6 Energy Flux Calculation

Energy fluxes were calculated by site‐ and treatment‐specific interaction networks, resulting in 116 soil food webs. We assumed energetic steady state and followed the general energy‐balance formulation proposed by Gauzens et al. (2019), where all energetic losses of a node are balanced by the assimilated energy it gains from consumption:

$$X_{i}+\sum F_{\mathrm{ij}}=\sum F_{\mathrm{ji}}\times e_{\mathrm{ij}}$$

with *X*_*i*_ the energetic losses of the species *i* (i.e., metabolic costs), *F*_*ij*_ the flux from species *i* to its consumer species *j*, *e*_*ij*_ the assimilation efficiency, which can either depend only on *i* (for predators), only on *j* (for detritivores), or on both (for omnivores) and F*ji* the fluxes from species *i* to consumer *j* as proportion, with the sum of proportions from all prey (*i*) species to *j* equaling 1. The proportions among nodes were derived from the constructed trophic network (see above).

Assimilation efficiency was calculated based on nitrogen concentration of basal resources and animal prey, according to the linear model formula proposed by Jochum et al. 2021:

$$\text{logit}\;e_{a}=0.471\times \text{nitrogen}\;\left(\%\right)-2.097$$

Assimilation efficiencies of plant (0.15), bacteria (0.73) and fungi (0.30) were taken from Junggebauer et al. (2025). Nitrogen content of animal prey of Collembola were extracted from measurements of bulk stable isotopes (Zhang, et al. 2025), resulting in an assimilation efficiency of 0.96. Assimilation efficiency for other animal prey groups and basal resources followed Potapov's method (Potapov 2022). The data of energy fluxes were converted from J to g year^−1^ m^−2^ (fresh biomass flux) roughly assuming 1 kg fresh biomass = 7 × 10^6^ J (Peters 1983).

### 2.7 Food‐Web Properties

Food‐web energetic properties were calculated for each of the 116 food webs. First, we computed the sum of energy fluxes of soil food webs along all trophic links to represent soil ecosystem multifunctionality. Trophic functions of animals were primarily linked to consumed food. Thus, herbivory represents the fluxes from living aboveground plant compartments (leaves and shoots) and living roots to animals, algivory represents the fluxes from algae to animals, wood and soil feeding represent the fluxes from wood and organic soil organic matter to animals, respectively, bactivory and fungivory represent the fluxes from bacteria and fungi to animals, respectively, and predation reflects the sum of fluxes from all animal nodes. These channels were used to inspect the energy redistribution in response to gap formation and deadwood addition.

To further analyse food web architecture, we calculated network connectance, cycling rate, turnover time, and hierarchy. Network connectance was calculated from the ratio of the number of realized links to all potential links among trophic groups, that is, network connectance = (number of links)/(number of groups)^2^ (Dunne et al. 2002; Pimm 1984). Carbon cycling rate was calculated as the ratio of bactivory to fungivory, assuming that bactivory represents faster and fungivory slower energy channeling (Rooney and McCann 2012). Turnover time was defined as the ratio of total biomass to total energy flux (transformed to fresh biomass flux) within the food web, representing the time needed for turnover of the existing biomass (Joly et al. 2020). Network hierarchy was calculated as the ratio of outgoing fluxes of basal resources to that of predation, suggesting a relative force between bottom‐up and top‐down control (Rosenberg et al. 2023).

### 2.8 Environmental Indicators

To identify potential drivers of the total energy flux through food webs associated with canopy gap formation and deadwood addition, we used site‐ and treatment‐specific data on climatic factors (i.e., temperature and moisture) and resource conditions (i.e., litter mass, microbial biomass carbon and fungi‐to‐bacteria ratio). As described above, we used the hourly measured temperature 20 cm above the soil averaged over spring in 2021. Moisture was measured gravimetrically by drying an aliquot of litter at 110°C for 24 h. Litter mass (dry weight) was measured gravimetrically from 20‐cm soil cores. Microbial biomass carbon in the litter layer was quantified using the substrate‐induced respiration (SIR) method by measuring the maximum initial respiratory response (MIRR; μg C g^−1^ dry weight soil and litter) after glucose addition (Anderson and Domsch 1978; Beck et al. 1997) with an automated electrolytic microrespirometer (Scheu 1992), thereby providing an estimate of the carbon contained in the living, metabolically responsive microbial biomass.

The fungi‐to‐bacteria ratio, derived from phospholipid fatty acids (PLFAs), was used as an indicator of the microbial community composition associated with litter. PLFAs were extracted from fresh litter (2 g) using a modified Bligh and Dyer method (Frostegård and Bååth 1996; Pollierer et al. 2015). Lipids were fractionated into neutral lipids, glycolipids, and phospholipids on silicic acid columns by sequential elution with chloroform, acetone, and methanol, respectively. The phospholipid fraction was subjected to mild alkaline methanolysis, and the resulting fatty acid methyl esters were analyzed using a GC‐FID Clarus 500 (PerkinElmer, Germany) equipped with an Elite 5 column (30 m × 0.32 mm inner diameter, 0.25 μm film thickness). Individual fatty acids were identified by comparing chromatographic retention times with commercial FAME and BAME standards, including standards for the methyl‐branched fatty acids me16:0, me17:0, and me18:0. The temperature program started at 60°C (1 min), increased by 30°C min^−1^ to 160°C, and subsequently by 3°C min^−1^ to 280°C. The injector temperature was 250°C, and helium was used as the carrier gas. PLFA abundances were expressed as nmol g^−1^ dry weight of litter. The PLFA 18:2ω6,9 was used as a fungal marker. Bacteria were represented by the sum of Gram^+^ (i15:0, a15:0, i16:0, i17:0, 10Me16:0, 10Me17:0, 10Me18:0) and Gram^−^ (cy17:0, cy19:0, 16:1ω7, and 18:1ω7) bacteria (Fanin et al. 2019; Zelles 1999). The fungi‐to‐bacteria ratio was calculated as the abundance of the fungal marker relative to the summed abundance of bacterial PLFAs.

### 2.9 Statistics

All statistical analyses were done in R 4.4.0 (R Core Team 2024). For total energy flux and biomass, linear mixed effect models (LMMs) were used with gap, deadwood and region (northern, central and southern Germany) and their interaction as fixed effects, and with treatments nested in 29 sites as random effect to account for spatial vicinity of samples. No significant spatial autocorrelation was detected in the model residuals based on Moran's *I* test for the total energy model (Moran's *I* = −0.001, *p* = 0.984). Because higher‐order interactions among gap, deadwood and region were not significant, we applied model‐based contrasts to estimate the effect size of gap (gap vs. closed canopy) and deadwood (with vs. without) across regions. Both biomass and total energy were log‐transformed to improve normality and homoscedasticity of model residuals as indicated by Levene's test. Forest type significantly affected total energy flux (*F*_6,18.9_ = 4.5, *p* = 0.005), but it did not significantly modify the effects of canopy gap formation or deadwood addition (forest type × gap: *F*_6,58.7_ = 1.8, *p* = 0.118; forest type × deadwood: *F*_6,58.7_ = 0.3, *p* = 0.943; forest type × gap × deadwood: *F*_6,58.7_ = 0.9, *p* = 0.442). Therefore, forest type was included as random factor in the main models to account for background variation while keeping the focus on the experimental treatments. For food‐web architecture properties (network connectance, cycling rate, turnover time, and hierarchy), we fitted LMMs with gap, deadwood and their interaction as fixed factors, and forest type and treatment nested in sites and regions as random factors.

To identify the potential drivers of the total energy flux through food webs associated with canopy gap formation and deadwood addition, we used site‐ and treatment‐specific data on climatic factors (i.e., temperature and moisture) and resources conditions (i.e., litter mass, microbial biomass carbon, and fungi‐to‐bacteria from litter). Given the observed strong interactions between gap and deadwood effects on total energy fluxes, we applied two piecewise structural equation models (piecewiseSEM) to explore environmental drivers separately for the gap (with vs. without) and gap + deadwood effects (with deadwood in canopy gap vs. the other three treatments, i.e., “control,” “gap,” and “deadwood”). We chose piecewiseSEM rather than standard SEM to accommodate the hierarchical design of the present study (Shipley 2009). Multiple linear models with site and region as random factors were combined and evaluated by a directional separation approach to test multivariate causal hypotheses (Shipley 2009). We modified models until the directional separation tests and Fisher's *C*‐test passed *p* > 0.05. Then, paths that caused problems of negative Chi‐squared, poor causal links (directional separation tests, *p* < 0.05) and poor model fit (Fisher's *C*‐test, *p* < 0.05) were subsequently removed.

Three sensitivity analyses were applied to improve the solidness for the effects of gap formation and deadwood addition on total energy flux. Due to uncertainty in the resource preferences assigned to trophic guilds, particularly omnivorous consumers, sensitivity analysis was applied to evaluate how this uncertainty affects our key results on total soil food‐web energy flux. We re‐ran the food‐web reconstructions and energy‐flux calculations across 11 scenarios, varying the relative importance of auxiliary resources (the “omnivory coefficient”) from 0 to 1 in steps of 0.25. For each scenario, we recomputed total food‐web energy flux for every plot and repeated the statistical analysis of treatment effects. For clarity and to focus on our management comparison, we report the sensitivity results for the two canopy‐gap treatments only (gap without deadwood, *gap*; and gap with deadwood, *gap + deadwood*), which quantify the effect sizes of deadwood retention under canopy opening (Figure S1). In addition, given the uncertainty associated with temperature measured 20 cm above the soil surface, a sensitivity analysis was conducted to evaluate whether lower forest‐floor temperatures may affect the estimated effects of gap and deadwood. We recalculated energy flux after reducing temperature by 1°C–5°C compared in 1°C increments. We then ran the LMMs for each of the temperature scenarios (Figure S2). To account for the potential bias arising from differences in earthworm sampling effort among regions, we reran the models after excluding individuals collected by mustard extraction at central and southern sites. The total energy flux was shifted after excluding the earthworms, but the major conclusions were not affected (Table S3).

The *nlme* and *lmerTest* packages were used to fit LMMs and ANOVAs, respectively (Bates et al. 2015; Pinheiro and Bates 2025), the *emmeans* package was used to calculate planned contrasts (Lenth 2022) and the *piecewiseSEM* package was used for piecewise SEM analysis (Shipley 2009). Results were visualized using the *ggplot2* package (Wickham 2016).

## 3 Results

### 3.1 Energy Fluxes and Biomass

Total food web energy fluxes differed significantly between treatments (Figures 1 and 2, Table S4). It generally decreased by −13.4% ± 9.6% in canopy gaps (*F*_1,76.6_ = 4.8, *p* = 0.032, Table S4). However, this depended on deadwood addition; in gaps with deadwood total energy fluxes were 37.4% higher than in gaps without deadwood (significant gap × deadwood interaction, *F*_1,76.6_ = 4.4, *p* = 0.040; Figure 1A, Table S4). This interaction was consistent across regions and prey categories (non‐significant gap × deadwood × region interaction, *F*_2,824.3_ = 1.9, *p* = 0.151; non‐significant gap × deadwood × prey category interaction, *F*_7,822.0_ = 1.3, *p* = 0.261; Table S5). The higher total energy fluxes in gaps with deadwood than in those without mainly contributed to the channels of fungivory (*p* = 0.024), wood feeding (*p* = 0.009), algivory (*p* = 0.003), litter feeding (*p* < 0.001) and predation (*p* = 0.035), mainly due to Collembola, Diplopoda, Chilopoda and Pseudoscorpionida benefitting from deadwood addition (Figures 1C and 2, and S2).

**FIGURE 1:**
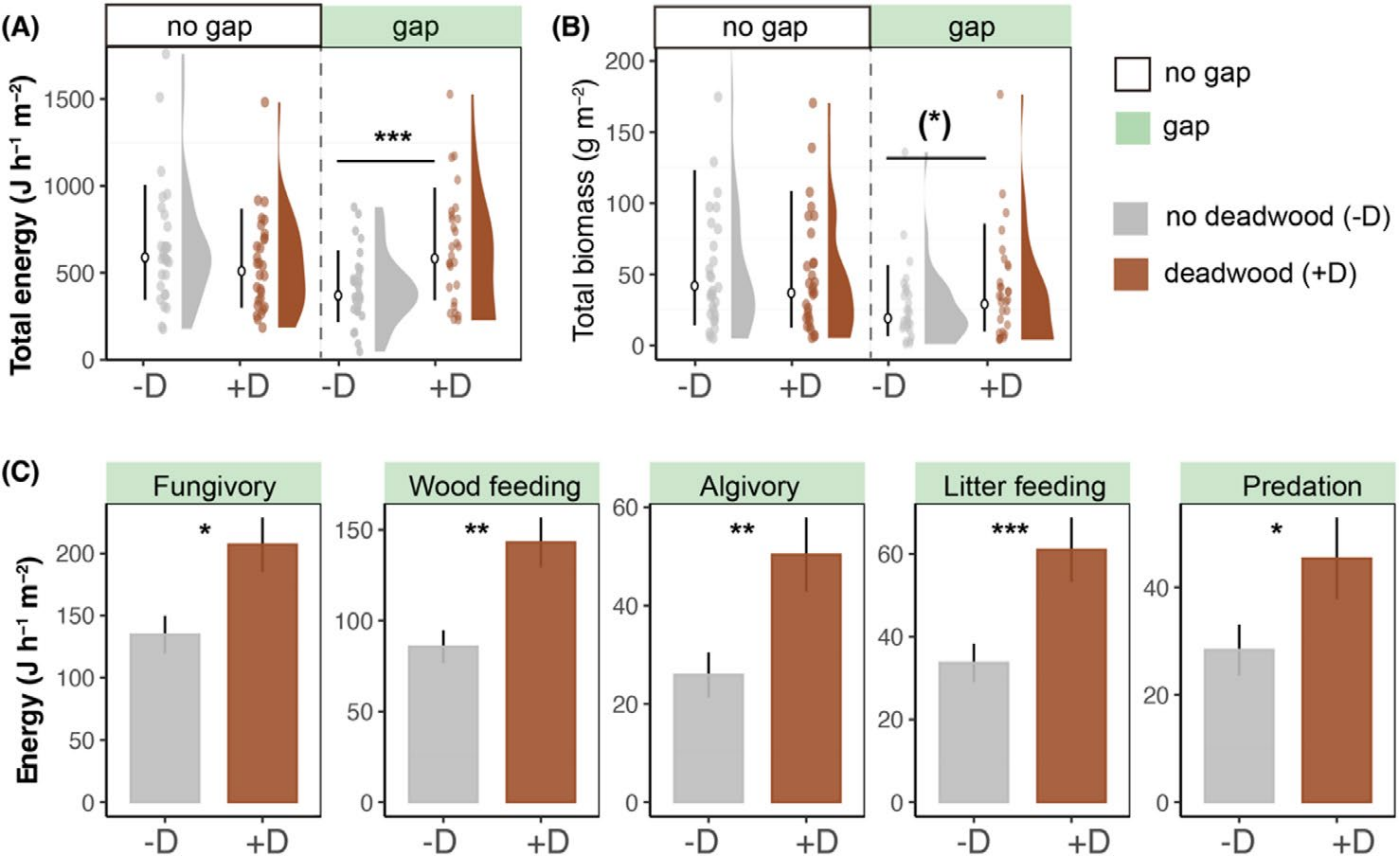
Effect of deadwood addition and canopy gap formation on soil food web energy fluxes and biomass. (A) Total energy flux of soil food webs (g year^−1^ m^−2^) across treatments combining canopy gap (no gap vs. gap) and deadwood (no deadwood [−D] vs. deadwood [+D]). (B) Total biomass of soil animals (g m^−2^). (C) Differences in energy flux through different pathways (fungivory, wood feeding, algivory, litter feeding, predation) between treatments with and without deadwood in canopy gaps. Violin plots show the distribution of plot‐level values; black points and error bars are means ±95% confidence intervals; asterisks indicate significant effects, (*) *p* < 0.1, *p < 0.05, **p < 0.01, and ***p < 0.001.

**FIGURE 2:**
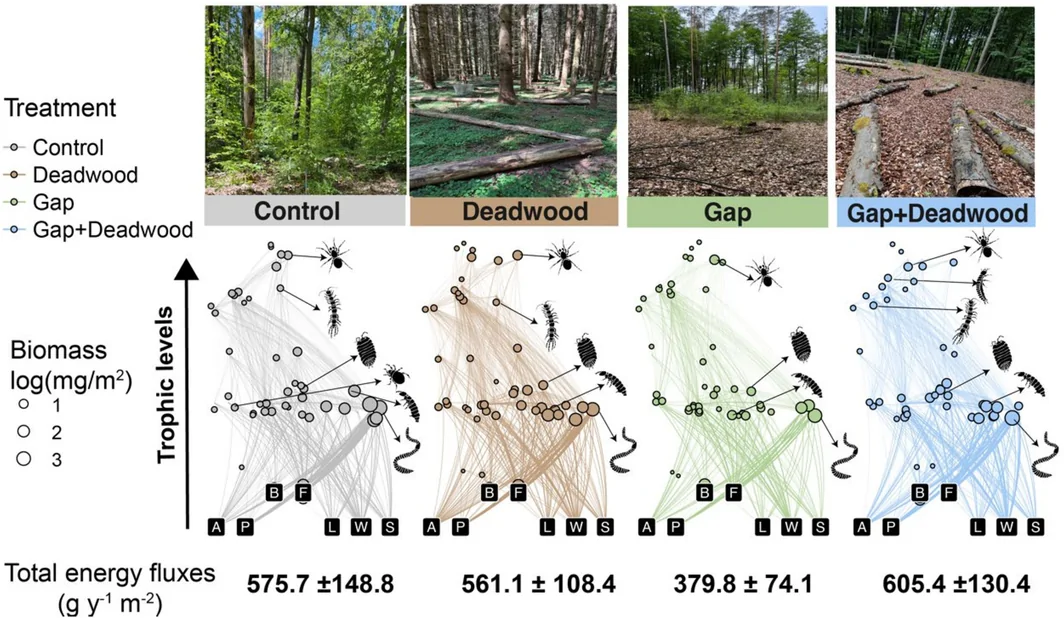
Experimental design and effects of canopy gap formation and deadwood addition on soil food‐web energy fluxes. Four treatments were established at each of 29 temperate forest sites (*n* = 116 soil food webs in total), that is, closed‐canopy (*Control*), deadwood addition (*Deadwood*), canopy gap formation (*Gap*) and both combined (*Gap + Deadwood*). Photographs at the top illustrate typical stand structure in each treatment. Network diagrams show reconstructed soil animal food webs; node size is proportional to the square root of biomass of each trophic group; black square nodes represent basal resources with A—algae, P—shoots or roots of living plants, B—bacteria, F—fungi, L—litter, W—deadwood, S—soil organic matter. Animal nodes are ordered horizontally according to the body mass and vertically according to the trophic level. Animal nodes channeling a major part of the animal energy flux (> 5%) in respective treatments are highlighted with silhouettes.

Similarly, total biomass significantly decreased in canopy gaps by 37.5% ± 21.9% compared to the average total biomass of 53,002 ± 12,813 mg m^−2^ in plots without gap (*F*_1,78_ = 12.9, *p* < 0.001), mainly due to Collembola and Pseudoscorpionida (Figure S3). Similar to total energy flux, gap effects on total biomass depended on deadwood (marginally significant gap × deadwood interaction, *F*_1,78_ = 3.1, *p* = 0.082; Figure 1B, Table S6).

Total energy flux and biomass varied strongly among geographical regions (*F*_2,26.1_ = 5.8, *p* = 0.008; *F*_2,78.0_ = 8.2, *p* = 0.002, respectively, Tables S4 and S6); both were significantly lower at northern than at central and southern sites (Figure S4). Additionally, canopy gaps decreased total energy fluxes in central and southern regions by −18.4% ± 29.9% and −34.1% ± 21.2% but increased those in northern regions by 28.3% ± 42.6% (significant gap × region interaction, *F*_2,76.6_ = 5.0, *p* = 0.009; Table S3, Figure S4). The positive deadwood effect on total energy flux was more prominent in the northern region, where it exceeded that in the central and southern region on average by 61.3% ± 49.5% (marginally significant deadwood × region interaction: *F*_2,76.6_ = 2.6, *p* = 0.080; Table S4).

### 3.2 Drivers of Energy Fluxes

Piecewise SEMs indicated that the reduced total energy flux in canopy gaps was primarily mediated by reduced amounts of litter (Figure 3). By contrast, the addition of deadwood to gaps altered microbial community structure, reflected by the increased fungi‐to‐bacteria ratio in the litter layer, which enhanced total energy fluxes.

**FIGURE 3:**
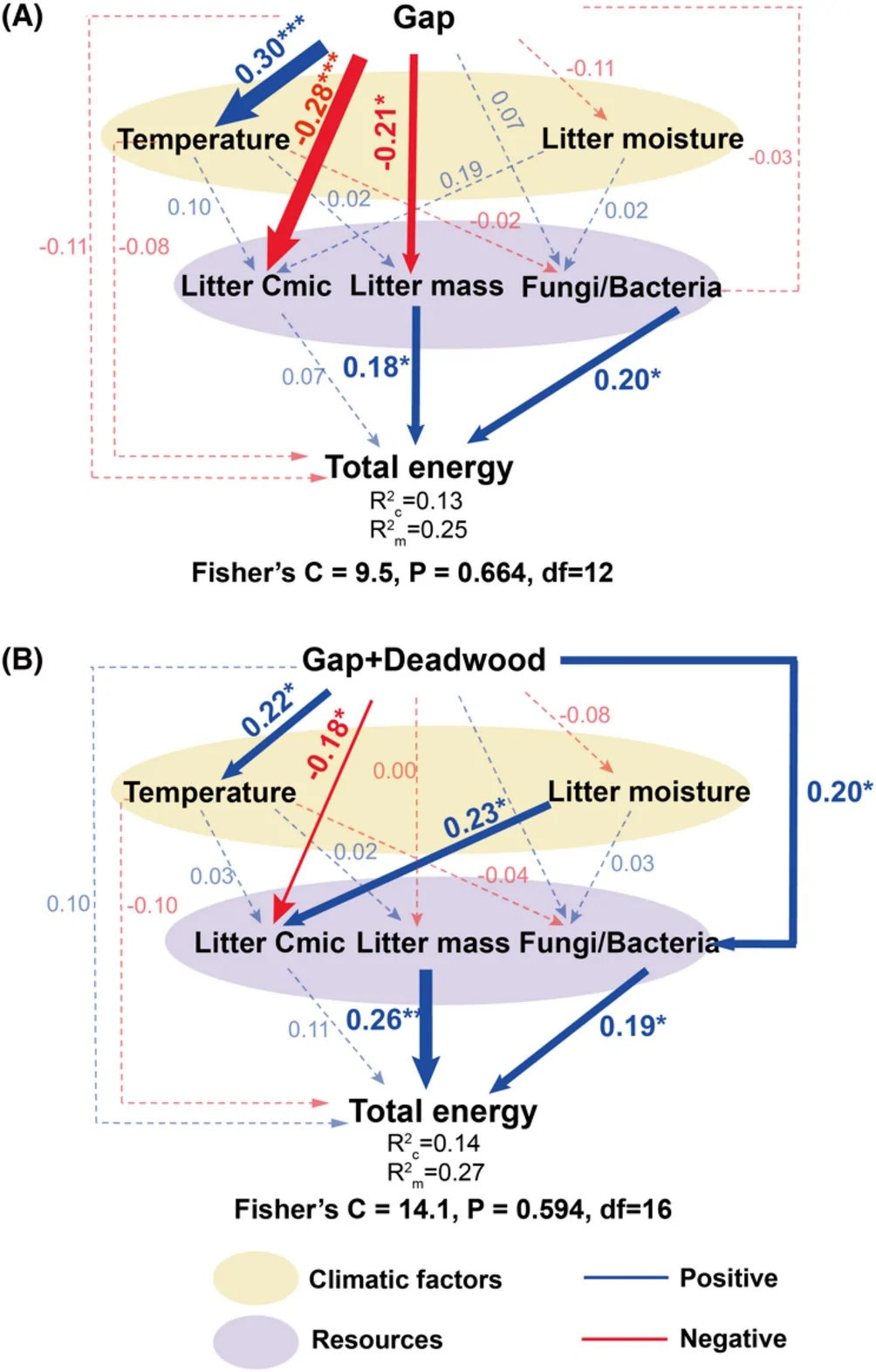
Structural equation models on factors driving total food web energy fluxes in forest canopy gaps without (A) and with deadwood (B). The conditional and marginal *R* squared, that is, *R*_c_^2^ and *R*_m_^2^, represent the proportion of variance explained by the variables without and with accounting for random effects of “region” and “site,” respectively. Red and blue colors of arrows indicate negative and positive effects, respectively. The type of arrows, that is, solid and dashed, indicate significant and non‐significant relationships, respectively. Numbers adjacent to arrows are standardized path coefficients; *p < 0.05, **p < 0.01, and ***p < 0.001. Fisher's *C*‐ and *p*‐values represent the goodness of fit; df, degrees of freedom.

### 3.3 Food‐Web Network Architecture

Canopy gap formation and deadwood addition restructured the soil food web (Figure 4, Table S7). In canopy gaps, network connectance was reduced (*F*_1,108.0_ = 8.1, *p* = 0.005) but the cycling rate was increased (*F*_1,82.0_ = 5.6, *p* = 0.020). However, the gap effect on both metrics depended on deadwood (significant gap × deadwood interaction, *F*_1,108.0_ = 4.8, *p* = 0.030 and *F*_1,82.1_ = 4.2, *p* = 0.031, respectively). Deadwood addition to canopy gaps restored network connectance and cycling rates to levels comparable to those in control plots. Canopy gap formation strongly decreased the turnover time (*F*_1,83.2_ = 17.1, *p* < 0.001), while it was increased along with deadwood added (marginally significant gap × deadwood interaction, *F*_1,83.2_ = 2.9, *p* = 0.093). Neither gap nor deadwood affected the network hierarchy.

**FIGURE 4:**
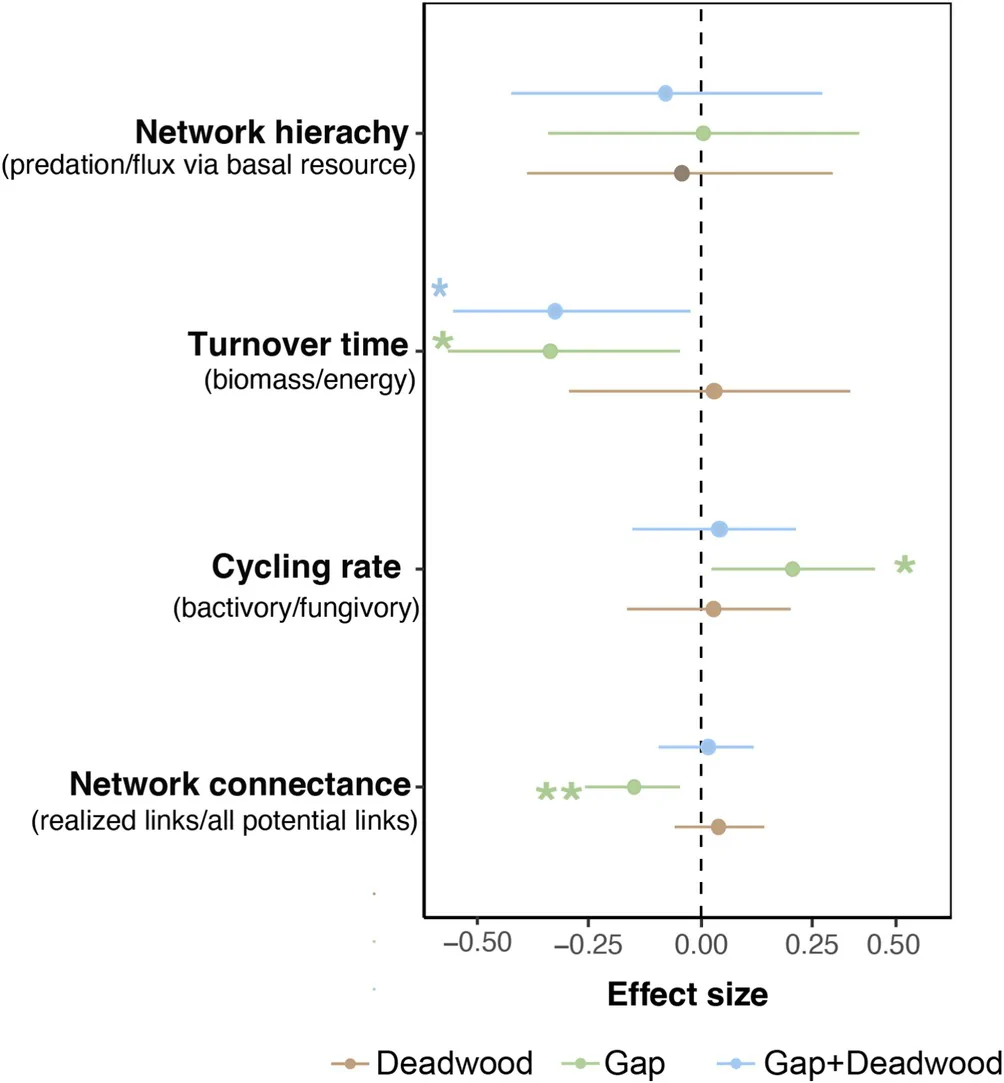
Effects of canopy gap formation and deadwood addition on network architecture indexes of soil food webs. Standardized effect sizes (mean ±95% confidence intervals) of deadwood (brown), gap (green) and gap + deadwood (blue) relative to the closed‐canopy control without deadwood addition. Four properties of network architecture are shown, that is, network hierarchy (ratio of predation to flux via basal resources), turnover time (biomass/energy), cycling rate (bacterivory/fungivory) and network connectance (realized links/all potential links). Asterisks indicate significant effects with *p < 0.05, and **p < 0.01.

## 4 Discussion

The full‐factorial design of our study allowed us to disentangle the ecological consequences of canopy opening from those of deadwood retention, thereby capturing contrasting outcomes of tree mortality resulting from forest management and natural disturbances. Canopy gaps without deadwood resemble managed forests where felled timber is removed, whereas canopy gaps with deadwood retention more closely represent naturally formed gaps, particularly those created by windthrow, where fallen trees remain in situ. Our findings therefore extend beyond managed forests to naturally disturbed forest ecosystems, while also providing direct implications for deadwood retention and post‐disturbance forest management.

### 4.1 Canopy Gaps Reduce Soil Food‐Web Energy Fluxes and Restructure Soil Food Webs

Canopy gap formation without deadwood reduced total energy flux of soil food webs, partly consistent with our first hypothesis and indicating a decline in belowground functioning. The reduction suggests that the formation of canopy gaps following timber harvesting reduces energy processing in belowground systems (Barnes et al. 2018; de Vries et al. 2013). The decline was mainly due to Isopoda and Diplura, whose biomass and associated energy flux were lower in canopy gaps than in closed‐canopy forests. This reduction was likely associated with the loss of plant‐derived resources following canopy opening including both root‐derived carbon and leaf litter. Food‐web studies have shown that living‐root inputs and detrital resources represent distinct energy channels supporting soil trophic functions (Pollierer et al. 2007; Zhou et al. 2023), and that shifts in their relative contribution can substantially alter soil food‐web functioning (Henneron et al. 2026). This is supported by our structural equation models, which showed that reduced litter mass mediated the loss of energy flux in forest gaps. Among the animal groups we studied, Isopoda (Scheu and Schaefer 1998; Zimmer 2002) and Diplura (Okuzaki et al. 2009) have shown to be particularly sensitive to these resources. Given the additional input of root‐derived detritus following tree removal, these belowground resources were apparently insufficient to compensate for the loss of aboveground leaf‐litter inputs after gap formation.

Additionally, in canopy gaps network connectance of soil food webs was reduced and the energy flux shifted from fungal‐based “slow” to bacterial‐based “fast” channels, as reflected by higher cycling rates. Combined with lower turnover time in canopy gaps, this points to depletion of carbon stocks in canopy gaps (de Vries et al. 2013; Joly et al. 2020). The dominance of fast bacterial energy channeling is consistent with conceptual and empirical work showing that open habitats, such as canopy gaps, clear‐cuts or production forests, favor bacterial pathways and increase bacterivory in soil food webs at the expense of fungal pathways (Fernández‐Martínez et al. 2014; Potapov et al. 2024). In our study, detrital inputs such as litter mass were strongly reduced, likely forcing decomposer communities to compensate for the shortage of these resources by intensifying exploitation of bacterial resources, resulting in a more rapid energy processing but potentially less resilient soil food webs (Rooney et al. 2006; Rooney and McCann 2012; de Vries et al. 2012). Supporting this conclusion, the decline in network connectance indicates that soil food webs in canopy gaps became less integrated, with energy flowing through a more restricted subset of interactions. Together, reduced total energy flux, the shift towards fast bacterial channels and lower connectance point to a simplified soil food web in forest canopy gaps, in which belowground functioning is weakened and fewer pathways are available to buffer further disturbances.

### 4.2 Deadwood Addition Mitigates Detrimental Effects of Forest Gaps on Soil Food‐Web Energy Channeling and Network Connectance

Deadwood addition generally increased the total energy flux through soil food webs, with this increase being stronger in canopy gaps than in closed forest, confirming our second hypothesis. The increase in total energy flux in canopy gaps with deadwood was mainly driven by detritivores (Collembola and Diplopoda) but also predators (Chilopoda and Pseudoscorpionida). Deadwood itself supplies energy‐rich substrates for decomposers, and these resources decompose particularly rapidly under warmer microclimatic conditions in canopy gaps (Griffiths et al. 2021; Perry et al. 2018), making them more accessible to detritivores such as Collembola and Diplopoda. At the same time, deadwood increases habitat heterogeneity and provides refuge sites and movement corridors, allowing predators to encounter and exploit prey more efficiently (Bauhus et al. 2018; Lassauce et al. 2011). This agrees with previous isotope‐based studies showing that deadwood shifts soil animal communities towards higher trophic positions and stronger predatory functioning (Fujii et al. 2023; Tuo et al. 2024; Zhang, Junggebauer, et al. 2025). Consistent with this dual role of deadwood as habitat and food resources, we found that fluxes of fungivory, wood feeding, and predation channels were enhanced in gaps with deadwood compared to gaps without deadwood.

Interestingly, the deadwood‐induced increase in energy fluxes through soil food webs in canopy gaps was more pronounced in the northern study region. The sites in the northern region are located on a young glacial landscape and are characterized by acidic soils of a pH of ~3.3. These base‐poor soils are considered ecologically vulnerable with limited buffering capacity (Pollierer et al. 2021; Thieme et al. 2019), with soil food webs being likely particularly sensitive to additional resources provided by deadwood.

Partly inconsistent with our third hypothesis, deadwood did not show a uniform main effect on energy cycling rates; instead, its influence was context dependent, and only counteracted the gap‐induced increase in cycling rate when added to canopy gaps. This likely reflects that the effects of deadwood decomposition on resource availability are most pronounced in forest gaps, as indicated by higher fungi‐to‐bacteria ratio in litter. Similarly, deadwood in canopy gaps restored network connectance of soil food webs to levels comparable to those under closed canopy. Higher connectance indicates that, for a given number of trophic groups, a larger fraction of potential feeding links is realized, so energy is channeled via a larger number of pathways in food webs (Dunne et al. 2002; Poisot and Gravel 2014). This may enhance resilience by providing alternative pathways for energy flow and compensatory dynamics when individual links are weakened or lost (Gardner and Ashby 1970; Pimm 1984; Rooney et al. 2006). In gaps with deadwood, this points to a more densely connected and integrated soil food web. This likely reflects both increased resource diversity and higher consumer diversity in gaps with deadwood, suggesting that deadwood adds new resource types (wood, deadwood‐associated fungi, microhabitats) and supports additional taxa, resulting in a larger number of trophic pathways (Lassauce et al. 2011; Stokland et al. 2012; Vodka et al. 2009). As a result, energy in forest gaps with deadwood is routed through a richer and more integrated interaction network, rather than being confined to few fast bacteria‐based pathways as in gaps without deadwood. Thus, deadwood not only compensates for energy losses in canopy gaps but also rebuilds a well‐connected soil food web, which is crucial for maintaining belowground ecosystem functioning after disturbances.

### 4.3 Management Implications

Based on our large‐scale, full‐factorial manipulative field experiment, our results suggest that the consequences of naturally formed canopy gaps may be less detrimental to belowground functioning when fallen trees remain in situ and identify deadwood retention in harvested canopy gaps as a tractable and important management lever to buffer declines in belowground functioning under increasingly frequent forest disturbances. However, as our experiment only captured early responses (2 years after the establishment of gaps and addition of deadwood), longer‐term monitoring is needed to assess whether these benefits persist or strengthen as deadwood decomposes and to determine how effects vary across decomposition stages. Nonetheless, our findings highlight deadwood retention as a practical strategy to maintain soil food‐web architecture and belowground functioning, thereby promoting long‐term carbon retention and healthy soil functioning in disturbance‐prone forests.

## 5 Conclusions

Canopy gaps reduced the total energy flux through and the biomass of soil animal food webs, shifted the energy flow from fungal‐based “slow” to bacterial‐based “fast” channels and lowered the network connectance of the animal food web, indicating simplified, fast‐cycling, and low‐retention animal food webs with weakened belowground functioning. By contrast, adding deadwood to canopy gaps increased total energy flux, particularly in the ecologically vulnerable northern region, enhanced fungivory, wood feeding and predation channels, and restored network connectance to levels similar to closed‐canopy forests. Together, these results identify deadwood as a key structural component and resource for soil animal food webs that mitigate detrimental effects of canopy gaps on the structure and functioning of belowground food webs. The results suggest that retaining deadwood in canopy gaps is key to buffer detrimental effects of forest disturbances on soil ecosystem functioning and helps in maintaining diverse trophic pathways under intensifying disturbance regimes.

## Author Contributions

**Yan Zhang:** methodology, software, data curation, formal analysis, visualization, writing – original draft, writing – review and editing. **Zheng Zhou:** conceptualization, supervision, writing – review and editing, visualization, methodology. **Gennuo Wang:** writing – review and editing. **Huimin Yi:** writing – review and editing. **Sarah L. Bluhm:** data curation, writing – review and editing. **Ajuan Zhang:** writing – review and editing. **Johanna E. Noske:** data curation, writing – review and editing. **Bin Xiao:** writing – review and editing, data curation. **Stefan Scheu:** conceptualization, methodology, supervision, writing – review and editing, funding acquisition. **Melanie M. Pollierer:** conceptualization, data curation, supervision, writing – review and editing, methodology. **André Junggebauer:** conceptualization, methodology, data curation, supervision, writing – review and editing.

## Funding

This study was funded by the Deutsche Forschungsgemeinschaft (DFG) in the framework of the Priority Program “Biodiversity Exploratories” (SCHE 376/38‐3). Zheng Zhou is partly funded by the Deutsche Forschungsgemeinschaft (DFG) (532858005).

## Conflicts of Interest

The authors declare no conflicts of interest.

## Supporting information


**Figure S1:** Sensitivity analysis of total soil food‐web energy flux under uncertainty in omnivory. Shown are percent changes in total energy flux (mean ±95% confidence intervals) across omnivory coefficients ranging from 0 to 1 (step = 0.25). Percent changes are expressed relative to the closed‐canopy control, and all effects shown are significant (*p* < 0.05). For clarity, only treatments with gaps (i.e., *gap*, *gap + deadwood*) are displayed.
**Figure S2:** Sensitivity analysis of total energy flux under uncertainty in temperature. Total energy flux was recalculated after uniformly reducing the temperature measured 20 cm above the soil surface by 1°C–5°C; 0°C represents the original temperature used in the analysis. Shown are the total energy flux (mean ± se) across temperature adjustments from −5°C to 0°C (step = 1). Results from the corresponding mixed‐effects models, using the same model structure as in Table S3, are shown on the right. For clarity, the effects of gap, deadwood, and their interaction are presented. Asterisks indicate statistical significance (*p < 0.05, **p < 0.01).
**Figure S3:** Responses of total energy fluxes of each soil animal group to treatment references to control (mean ±95% confidence intervals). *p < 0.05, **p < 0.01, ***p < 0.001.
**Figure S4:** Responses of total biomass of each soil animal group to treatment references to ‘control’ plots (mean ±95% confidence intervals). *p < 0.05, **p < 0.01, ***p < 0.001.
**Figure S5:** Total energy flux (a) and total biomass (b) in forest soil food webs of northern (SEW), central (HEW), and southern (AEW) Germany (a). Mean ±95% confidence intervals. Different letters suggest significance at *p* < 0.05.
**Table S1:** Allometric equations for estimating dry weight (DW) and fresh weight (WW) of studied invertebrate groups. Either body length (L) or both length and width (LW) are used in the power equations. The data are from various primary sources (given in the table, see also supplementary reference), secondary sources (Ehnes et al. 2011, cited in the main manuscript) as well as original data from this study.
**Table S2:** Feeding preferences, prey and predator functional traits, and life stage for high‐rank taxonomic groups of soil animals. Resources include eight types: living plant (P), algae (A), leaf and root litter (L), wood litter (W), soil organic matter (S), bacteria (B), fungi (F), and fauna (Fa). Numbers for resources indicate trophic strength with “1” for main food, “0.5” for supplementary food, and “0” for no trophic link at all. Numbers for functional traits indicate functional strength with “1” for strong, “0.7” medium, and “0.4” for weak. Numbers for above, epidaphic (epi), hemiedaphic (hemi) and euedaphic (eu) indicate relative frequency for spatial distribution, with “1” for very often, “0.5” for often, “0” for rare.
**Table S3:** F‐ and *p*‐values of linear models on the effect of canopy gaps, deadwood addition, and their interactions on total energy fluxes. Significant effects are given in bold, *p < 0.05, **p < 0.01; DenDF, denominator degrees of freedom; NumDF, numerator degrees of freedom.
**Table S4:** F‐ and *p*‐values of linear models on the effect of canopy gaps, deadwood addition, regions, and their interactions on total energy fluxes. Significant effects are given in bold, (*) *p* < 0.1, *p < 0.05, **p < 0.01; DenDF, denominator degrees of freedom; NumDF, numerator degrees of freedom.
**Table S5:** F‐ and *p*‐values of linear models on the effect of canopy gaps, deadwood addition, regions, prey category, and their interactions on total energy fluxes. Significant effects are given in bold, *p < 0.05, **p < 0.01, ***p < 0.001; DenDF, denominator degrees of freedom; NumDF, numerator degrees of freedom.
**Table S6:** F‐ and *p*‐values of linear models on the effect of canopy gaps, deadwood addition, regions, and their interactions on total biomass. Significant effects are given in bold, (*) *p* < 0.1, *p < 0.05, **p < 0.01, ***p < 0.001; DenDF, denominator degrees of freedom; NumDF, numerator degrees of freedom.
**Table S7:** F‐ and *p*‐values of linear models on the effect of canopy gaps, deadwood addition, regions, and their interactions on network architecture, that is, network connectance, cycling rate, turnover time, and network hierarchy. Significant effects are given in bold, (*) *p* < 0.1, *p < 0.05, ***p < 0.001; DenDF, denominator degrees of freedom; NumDF, numerator degrees of freedom.

## Data Availability

The datasets and code supporting the soil food web reconstruction are available via Figshare (https://doi.org/10.6084/m9.figshare.31440835).
