# Supplementary material for: Deadwood Mitigates Declines in Soil Food‐Web Energy Fluxes in Canopy Gaps
Source: Glob Chang Biol. 2026 Nov 8;32(11):e71197. doi: 10.1111/gcb.71197 (PMC13745870; doi:10.1111/gcb.71197)
Supplement: Supplementary file 1 [file GCB-32-e71197-s001.zip › supinfo/gcb71197-sup-0001-TableS1-S7-FigureS1-S5@R1-supplementary information-clean.docx]

**Supplementary information**

**Title: Deadwood mitigates declines in soil food-web energy fluxes in canopy gaps**

Yan Zhang^1^, Zheng Zhou^1,2*^, Gennuo Wang^1^, Huimin Yi^3,4^, Sarah L. Bluhm^1^, Ajuan Zhang^5^, Johanna E. Noske^1^, Bin Xiao^1^, Stefan Scheu^1,6#^, Melanie M. Pollierer^1,7#^, André Junggebauer^1#^

^1^J.F. Blumenbach Institute of Zoology and Anthropology, University of Göttingen, Göttingen, Germany

^2^Ecology of Tropical Agricultural Systems, University of Hohenheim, Stuttgart, Germany

^3^German Centre for Integrative Biodiversity Research (iDiv) Halle-Jena-Leipzig, Puschstraße, Leipzig, Germany

^4^Institute of Biology, Leipzig University, Puschstraße, Leipzig 04103, Germany

^5^Senckenberg Museum of Natural History Görlitz, Department of Soil Zoology, Görlitz, Germany

^6^Centre of Biodiversity and Sustainable Land Use, University of Göttingen, Göttingen, Germany

^7^Julius Kühn-Institute, Quedlinburg, Germany

*Corresponding author [zzhou@uni-goettingen.de](mailto:zzhou@uni-goettingen.de)

\# Equal contribution

**Table S1**. Allometric equations for estimating dry weight (DW) and fresh weight (WW) of studied invertebrate groups. Either body length (L) or both length and width (LW) are used in the power equations. The data are from various primary sources (given in the table, see also supplementary reference), secondary sources (Potapov et al. 2019, Ehnes et al. 2011, cited in the main manuscript) as well as original data from this study.

**Table S2** Feeding preferences, prey and predator functional traits and life stage for high-rank taxonomic groups of soil animals. Resources include eight types: living plant (P), algae (A), leaf and root litter (L), wood litter (W), soil organic matter (S), bacteria (B), fungi (F), and fauna (Fa). Numbers for resources indicate trophic strength with “1” for main food, “0.5” for supplementary food, and “0” for no trophic link at all. Numbers for functional traits indicate functional strength with “1” for strong, “0.7” medium, and “0.4” for weak. Numbers for above, epidaphic (epi), hemiedaphic (hemi) and euedaphic (eu) indicate relative frequency for spatial distribution, with “1” for very often, “0.5” for often, “0” for rare.

**Table S3** F- and *P*-values of linear models on the effect of canopy gaps, deadwood addition and their interactions on total energy fluxes. Significant effects are given in bold, * *P* < 0.05, ** *P* < 0.01; NumDF, numerator degrees of freedom; DenDF, denominator degrees of freedom.

|  | NumDF | DenDF | F value | *P value* |
| --- | --- | --- | --- | --- |
| Gap (G) | 1 | 82.4 | **5.1** | **0.027*** |
| Deadwood (D) | 1 | 82.4 | **7.4** | **0.008**** |
| G × D | 1 | 82.4 | **4.2** | **0.043*** |

**Table S4** F- and *P*-values of linear models on the effect of canopy gaps, deadwood addition, regions and their interactions on total energy fluxes. Significant effects are given in bold, (*) *P* < 0.1, * *P* < 0.05, ** *P* < 0.01; NumDF, numerator degrees of freedom; DenDF, denominator degrees of freedom.

|  | NumDF | DenDF | F value | *P* value |
| --- | --- | --- | --- | --- |
| Gap (G) | 1 | 76.6 | **4.8** | **0.032*** |
| Deadwood (D) | 1 | 76.6 | **7.2** | **0.001**** |
| Region (R) | 2 | 26.1 | **5.8** | **0.008**** |
| G × D | 1 | 76.6 | **4.4** | **0.040*** |
| G × R | 2 | 76.6 | **5.0** | **0.009**** |
| D × R | 2 | 76.6 | **2.6** | **0.080 (*)** |
| G × D × R | 2 | 76.6 | 0.6 | 0.551 |

|  | NumDF | DenDF | F value | *P* value |
| --- | --- | --- | --- | --- |
| Gap (G) | 1 | 824.4 | **9.9** | **0.002**** |
| Deadwood (D) | 1 | 824.4 | **25.6** | **<0.001***** |
| Region (R) | 2 | 25.9 | **13.9** | **0.006**** |
| Prey category (P) | 7 | 822.0 | **106.4** | **<0.001***** |
| G × D | 1 | 824.4 | **16.9** | **<0.001***** |
| G × R | 2 | 824.3 | **19.6** | **<0.001***** |
| G × P | 7 | 822.0 | 1.0 | 0.080 |
| D × R | 2 | 824.3 | **9.6** | **<0.001***** |
| D × P | 7 | 822.0 | 0.4 | 0.890 |
| R × P | 14 | 822.0 | 5.1 | **<0.001***** |
| G*D*R | 2 | 824.3 | 1.9 | 0.151 |
| G*D*P | 7 | 822.0 | 1.3 | 0.261 |

**Table S5** F- and *P*-values of linear models on the effect of canopy gaps, deadwood addition, regions, prey category and their interactions on total energy fluxes. Significant effects are given in bold, * *P* < 0.05, ** *P* < 0.01, *** *P* < 0.001; NumDF, numerator degrees of freedom; DenDF, denominator degrees of freedom.

**Table S6** F- and *P*-values of linear models on the effect of canopy gaps, deadwood addition, regions and their interactions on total biomass. Significant effects are given in bold, (*) *P* <0.1, * *P* < 0.05, ** *P* < 0.01, *** *P* < 0.001; NumDF, numerator degrees of freedom; DenDF, denominator degrees of freedom.

|  | NumDF | DenDF | F value | *P value* |
| --- | --- | --- | --- | --- |
| Gap (G) | 1 | 78 | **12.9** | **<0.001***** |
| Deadwood (D) | 1 | 78 | 0.7 | 0.398 |
| Region (R) | 2 | 78 | **8.2** | **0.002**** |
| G × D | 1 | 78 | **3.1** | **0.082(*)** |
| G × R | 2 | 78 | 1.8 | 0.164 |
| D × R | 2 | 78 | 0.1 | 0.901 |
| G × D × R | 2 | 78 | 0.4 | 0.655 |

**Table S7** F- and *P*-values of linear models on the effect of canopy gaps, deadwood addition, regions and their interactions on network architecture, i.e., network connectance, cycling rate, turnover time and network hierarchy. Significant effects are given in bold, (*) *P* <0.1, * *P* < 0.05, *** *P* < 0.001; NumDF, numerator degrees of freedom; DenDF, denominator degrees of freedom.

|  |  | NumDF | DenDF | F value | *P value* |
| --- | --- | --- | --- | --- | --- |
| Connectance | Gap (G) | 1 | 108.0 | **8.1** | **0.005**** |
|  | Deadwood (D) | 1 | 108.0 | **8.9** | **0.003**** |
|  | G × D | 1 | 108.0 | **4.8** | **0.030*** |
|  | | | | | |
| Cycling rate | G | 1 | 82.0 | **5.6** | **0.020*** |
|  | D | 1 | 82.1 | 1.3 | 0.249 |
|  | G × D | 1 | 82.1 | **4.2** | **0.031*** |
|  | | | | | |
| Turnover time | G | 1 | 83.2 | **17.1** | **<0.001***** |
|  | D | 1 | 83.2 | 0.9 | 0.348 |
|  | G × D | 1 | 83.2 | **2.9** | **0.093(*)** |
|  | | | | | |
| Hierarchy | G | 1 | 83.6 | 0.3 | 0.788 |
|  | D | 1 | 83.7 | 0.6 | 0.661 |
|  | G × D | 1 | 83.7 | 0.9 | 0.463 |

**Supplementary figures**

**Figure S1** Sensitivity analysis of total soil food-web energy flux under uncertainty in omnivory. Shown are percent changes in total energy flux (mean ± 95% confidence intervals) across omnivory coefficients ranging from 0 to 1 (step = 0.25). Percent changes are expressed relative to the closed-canopy control, and all effects shown are significant (*P* < 0.05). For clarity, only treatments with gaps (i.e., *gap*, *gap+deadwood*) are displayed.

**Figure S2** Sensitivity analysis of total energy flux under uncertainty in temperature. Total energy flux was recalculated after uniformly reducing the temperature measured 20 cm above the soil surface by 1-5 °C; 0°C represents the original temperature used in the analysis. Shown are the total energy flux (mean ± se) across temperature adjustments from -5 to 0 °C (step = 1). Results from the corresponding mixed-effects models, using the same model structure as in table S3, are shown on the right. For clarity, the effects of gap, deadwood and their interaction are presented. Asterisks indicate statistical significance (* *P* < 0.05, ** *P* < 0.01)

**Figure S3** Responses of total energy fluxes of each soil animal group to treatment references to control (mean ± 95% confidence intervals). * *P* < 0.05, ***P* < 0.01, *** *P* < 0.001.

**Figure S4** Responses of total biomass of each soil animal group to treatment references to ‘control’ plots (mean ± 95% confidence intervals). * *P* < 0.05, ***P* < 0.01, *** *P* < 0.001.

**Figure S5** Total energy flux (a) and total biomass (b) in forest soil food webs of northern (SEW), central (HEW) and southern (AEW) Germany (a). Mean ± 95% confidence intervals. Different letters suggest significance at *P* < 0.05
